# Spatial metabolomics informs the use of clinical imaging for improved detection of cribriform prostate cancer

**DOI:** 10.1073/pnas.2502423122

**Published:** 2025-06-23

**Authors:** Nikita Sushentsev, Gregory Hamm, Roido Manavaki, Mary A. McLean, Jonathan Birchall, Dmitry Soloviev, David Y. Lewis, Luigi Aloj, Lucy Flint, Aleksandr Zakirov, Ian G. Mills, Vincent J. Gnanapragasam, Anne Y. Warren, Simon T. Barry, Richard J. A. Goodwin, Ferdia A. Gallagher, Tristan Barrett

**Affiliations:** ^a^Department of Radiology, Addenbrooke’s Hospital and University of Cambridge, Cambridge Biomedical Campus, Cambridge CB2 0QQ, United Kingdom; ^b^Integrated BioAnalysis, Clinical Pharmacology and Safety Sciences, R&D, AstraZeneca, Cambridge CB2 0AA, United Kingdom; ^c^Lewis Group, School of Cancer Sciences, University of Glasgow, Glasgow G61 1BD, United Kingdom; ^d^Department of Clinical Neurosciences, University of Cambridge, Cambridge CB2 0QQ, United Kingdom; ^e^Nuffield Department of Surgical Sciences, University of Oxford, Oxford OX3 9DU, United Kingdom; ^f^Prostate Cancer Centre of Excellence, Patrick G. Johnston Centre for Cancer Research, Queen’s University Belfast, Belfast BT9 7AE, United Kingdom; ^g^Department of Biomedicine, Aarhus University, Aarhus DK-8000, Denmark; ^h^Department of Urology, Cambridge University Hospitals National Health Service Foundation Trust, Cambridge CB2 0QQ, United Kingdom; ^i^Cambridge Urology Translational Research and Clinical Trials Office, Addenbrooke’s Hospital, Cambridge Biomedical Campus, Cambridge CB2 0QQ, United Kingdom; ^j^Department of Pathology, Cambridge University Hospitals National Health Service Foundation Trust, Cambridge CB2 0QQ, United Kingdom; ^k^Bioscience, Discovery, Oncology Research and Development, AstraZeneca, Cambridge CB2 0AA, United Kingdom

**Keywords:** prostate cancer, cancer metabolism, MRI, nuclear medicine, spectroscopy

## Abstract

Cribriform prostate cancer (crPCa) is associated with poor clinical outcomes, yet its accurate detection remains challenging due to the poor sensitivity of standard-of-care diagnostic tools. Here, we use untargeted spatial metabolomics to identify fatty acid biosynthesis as a key metabolic pathway enriched in crPCa epithelium. We also show that imaging tumor lipid metabolism using [1-^11^C]acetate PET/CT and proton magnetic resonance spectroscopy differentiates cribriform from noncribriform intermediate-risk prostate cancers in two prospective patient cohorts. These findings support the feasibility of using clinical metabolic imaging techniques as adjunctive tools for improving crPCa detection in clinical practice, with prospective studies in larger cohorts warranted to obtain definitive results.

## Significance

Cribriform prostate cancer is a specific disease phenotype known for its high potential of transforming into incurable or even lethal disease. As conventional diagnostic methods tend to miss this disease subtype, identifying biologically informed methods for its early detection is important. Here, we show that one such method could be the imaging of tumor lipid metabolism using clinical imaging techniques: [1-^11^C]acetate positron emission tomography/computed tomography (PET/CT) and proton magnetic resonance spectroscopy. Both techniques, which are available to clinicians, showed promise for distinguishing between cribriform and noncribriform tumors in patients with biopsy-proven prostate cancer.

Cribriform prostate cancer (crPCa), manifesting in the form of either intraductal carcinoma or invasive cribriform carcinoma, independently predicts biochemical recurrence, metastasis, and disease-specific mortality (1). Early detection of crPCa is therefore critical, as reflected in its mandatory reporting in diagnostic biopsies (2, 3) and exclusion of patients with any crPCa from active surveillance in European clinical guidelines (4). However, MRI-targeted biopsy shows poor sensitivity for detecting crPCa (5), likely due to overlapping apparent diffusion coefficient (ADC) and dynamic contrast-enhanced features between cribriform and noncribriform lesions (6). To address this, we used untargeted spatial metabolomics to identify fatty acid biosynthesis as a key metabolic pathway distinguishing crPCa from noncribriform disease in radical prostatectomy (RP) samples. Drawing on previous research demonstrating the advantage of PCa detection using [1-^11^C]acetate PET/CT over routine 2-fluoro-2-deoxy-D-glucose PET (7–9), we then assessed the ability of [1-^11^C]acetate PET/CT and ^1^H magnetic resonance spectroscopic imaging (^1^H-MRSI) to noninvasively differentiate cribriform from noncribriform intermediate-risk tumors, compared to standard ADCratio assessment.

## Results

**Fig. 1.**
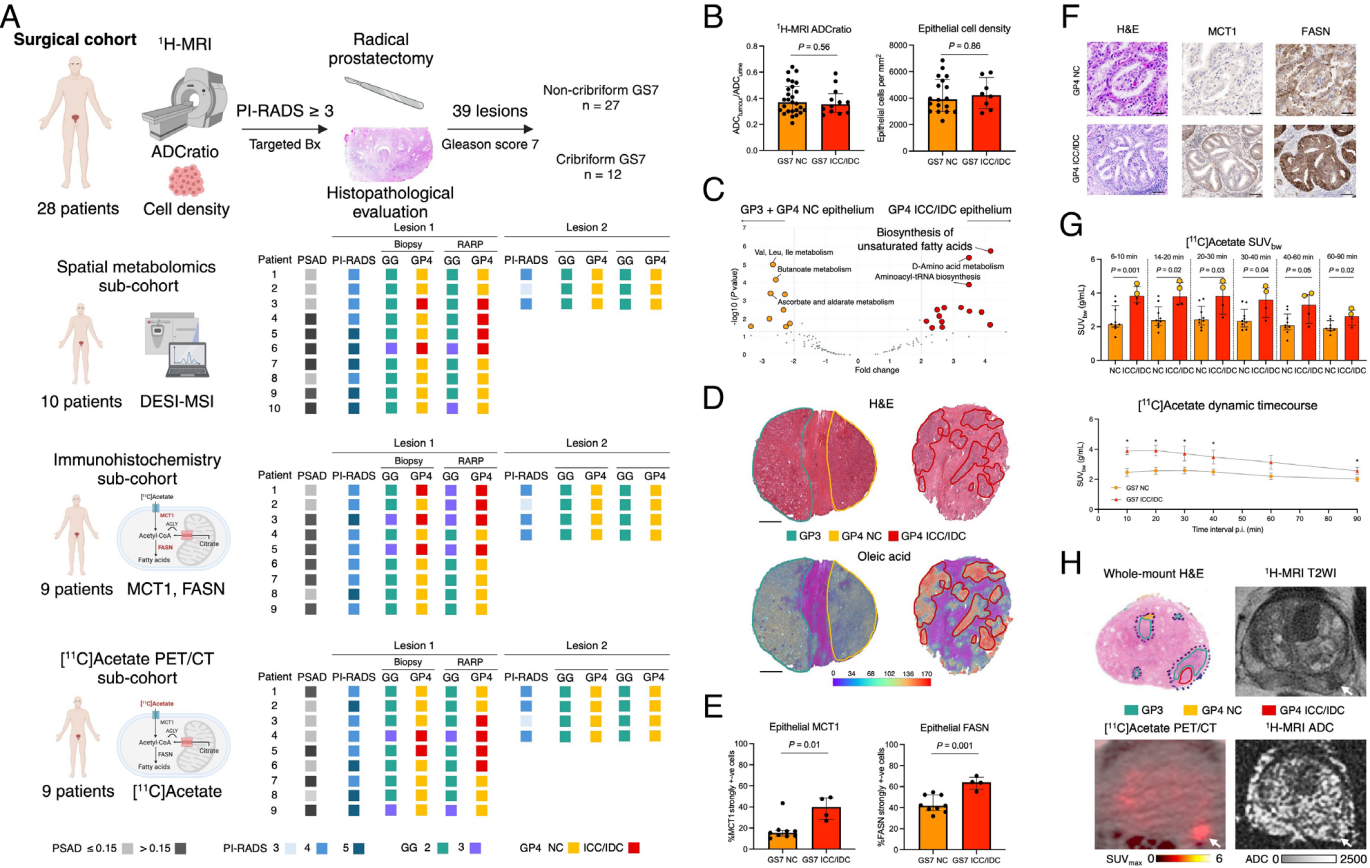
Clinical metabolic imaging for crPCa detection in surgical patients. (*A*) Study flowchart including the constituent subcohorts with matching clinico-pathologic characteristics. (*B*) Comparison of ^1^H-MRI tumor-to-urine ADCratio and digital pathology-derived epithelial cell density between cribriform and noncribriform GS7 tumors in the surgical cohort. (*C*) Metabolic pathway enrichment analysis of cribriform (red) and noncribriform (yellow) epithelial regions from the spatial metabolomics subcohort, with top three pathways labeled for each morphological entity; for the same plot featuring individual metabolites, see *SI Appendix*. (*D*) Representative hematoxylin and eosin (H&E) and DESI-MSI slides from specimens containing cribriform and noncribriform morphologies derived from the same patient from the spatial metabolomics subcohort; scale bars denote 2 mm. (*E*) Comparison of the percentage of cells with strongly positive MCT1 and FASN immunoexpression between cribriform and noncribriform tumors from the IHC subcohort. (*F*) Representative H&E and IHC images from the IHC subcohort; scale bars denote 50 μm. (*G*) Comparison of [1-^11^C]acetate SUV_bw_ between cribriform and noncribriform tumors from the [1-^11^C]acetate PET/CT subcohort; yellow dots denote SUV_bw_ values for the two cribriform lesions misclassified at diagnostic biopsy. (*H*) Representative whole-mount H&E, ^1^H-MRI, and [1-^11^C]acetate PET/CT images from a patient harboring [1-^11^C]acetate PET/CT-visible cribriform lesion in the left peripheral zone (white arrow) and [1-^11^C]acetate PET/CT-occult noncribriform transition zone tumor.

In a primary cohort of 28 PCa patients with MRI-visible biopsy-proven disease, RP identified 39 Gleason score 7 (GS7) tumors, comprising 27 noncribriform and 12 cribriform lesions based on the dominant Gleason pattern 4 (GP4) phenotype (Fig. 1*A*). MRI-targeted biopsy identified only 7/12 cribriform tumors, with a sensitivity of 58%, consistent with prior reports (5). ADCratio and digital pathology-derived epithelial cell density showed no significant differences between cribriform and noncribriform tumors (Fig. 1*B*).

Using spatially resolved desorption electrospray ionization mass spectrometry imaging (DESI-MSI) in one subcohort (Fig. 1*A*), we found that unsaturated fatty acid biosynthesis was significantly enriched in cribriform epithelium (Fig. 1 *C* and *D*), with 7 out of the 10 most distinctively enriched metabolites being unsaturated fatty acids (*SI Appendix*). Immunohistochemistry in a second subcohort showed significant overexpression of the plasma membrane transporter for acetate (monocarboxylate transporter 1; MCT1) and fatty acid synthase (FASN) in cribriform GS7 tumors compared to noncribriform lesions (Fig. 1 *E* and *F*). Consistently, [1-^11^C]acetate PET/CT demonstrated an average 1.5-fold increase in body-weight-normalized standardized uptake values (SUVbw) in cribriform lesions, sustained up to 60 min postinjection (*P* < 0.05; Fig. 1 *G* and *H*). Notably, two cribriform tumors misclassified on biopsy compared to prostatectomy had the highest [1-^11^C]acetate uptake (Fig. 1*G*).

**Fig. 2.**
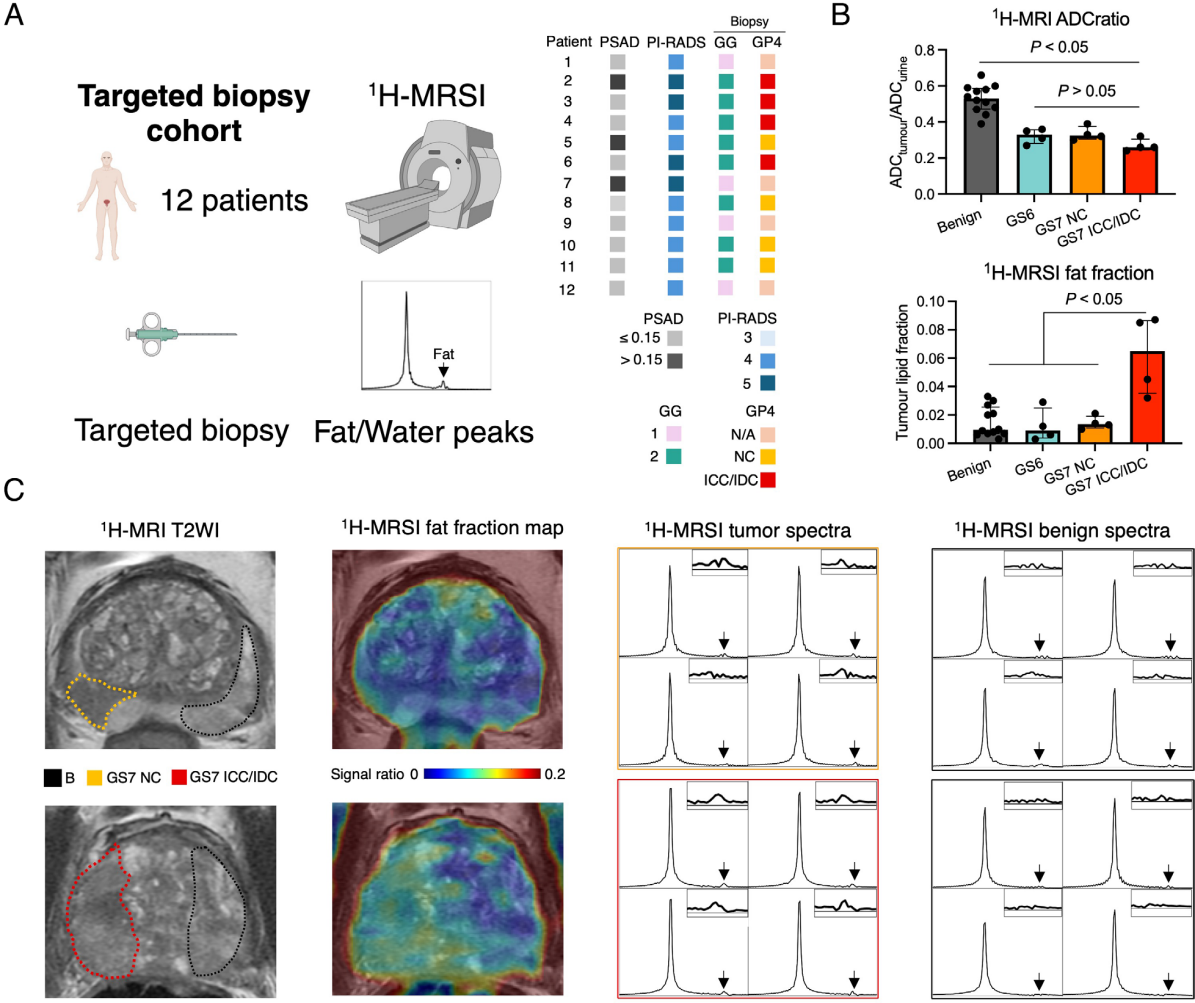
Clinical fat-water ^1^H-MRSI for crPCa detection in patients undergoing MRI-targeted biopsy. (*A*) Cohort description. (*B*) Comparison of ^1^H-MRI tumor-to-urine ADCratio and ^1^H-MRSI fat fraction between areas of histologically benign prostate, along with GS6, GS7 noncribriform, and GS7 cribriform tumors. (*C*) Representative T_2_-weighted images (T2WI) from patients with noncribriform (*Top*) and cribriform (*Bottom*) GS7 tumors with black, yellow, and red dotted areas denoting benign, noncribriform, and cribriform regions-of-interests (ROIs) used for extracting ^1^H-MRI tumor-to-urine ADCratio and ^1^H-MRSI fat fraction data. The panel also presents fused T2WI and ^1^H-MRSI fat fraction maps from the same patients, along with representative 2 × 2 spectra from voxels within the outlined benign and tumor ROIs; lipid peaks are denoted by black arrows, with boxes in the right top corner of spectra presenting their magnified images. To aid visualization, these spectra have thicker lines and are black in color compared to the raw spectra presented in *SI Appendix*.

To complement the use [1-^11^C]acetate PET/CT as a sensitive nuclear medicine technique which may not be routinely available outside centers with sufficient capacity for in-house [1-^11^C]acetate production, we also explored ^1^H-MRSI as an alternative clinical lipid imaging technique with high metabolite specificity and the potential to be added to the standard-of-care prostate MRI protocol. To do so, we performed ^1^H-MRSI as part of routine imaging in a prospective cohort of 12 PCa patients who underwent ^1^H-MRSI prior to an MRI-targeted biopsy (Fig. 2*A*). ^1^H-MRSI-derived fat fraction showed an almost fivefold increase in cribriform GS7 lesions compared to all other tissue types, including GS7 noncribriform tumors, GS6 tumors, and histologically benign regions (*P* < 0.05; Fig. 2 *B* and *C*). These findings aligned with the DESI-MSI results, while the ADCratio showed no distinction between the three tumor subtypes (Fig. 2*B*).

## Discussion

This study demonstrates the feasibility of using two clinical metabolic imaging techniques—[1-^11^C]acetate PET/CT and ^1^H-MRSI—to improve noninvasive detection of crPCa by targeting its distinct metabolic features. Unlike our previous work, where DESI-MSI analysis was limited to just three metabolic pathways (10), here we leverage the full list of detected metabolites to confirm that increased fatty acid biosynthesis is among the most distinctive metabolic features of crPCa epithelium. As more in-depth studies of crPCa metabolism are conducted, the findings presented here warrant prospective multicenter validation in larger cohorts to assess the integration of metabolic imaging into routine clinical practice for improved crPCa detection.

## Materials and Methods

Prior to surgery, all patients from the spatial metabolomics, immunohistochemistry, and [1-^11^C]acetate PET/CT subcohorts provided written informed consent for participation in the DIAMOND (03/018), MISSION-Prostate (16/EE/0205), and APtITuDE (CUH/15/EE/0213) studies approved by the National Research Ethics Service (NRES) Committee East of England, Cambridge South. APtITuDE study was also approved by the Administration of Radioactive Substances Advisory Committee (certificate reference RPC/83/400/33606). All patients from the targeted biopsy cohort provided written informed consent for adding ^1^H-MRSI to their standard-of-care prostate MRI protocol as part of the prospective study approved by NRES East of England, Cambridge South (16/EE/0346). ^1^H-MRI ADCratio and epithelial cell density quantification methods have been described previously (10, 11). Detailed DESI-MSI and immunohistochemistry acquisition and analysis protocols are provided in the original cohort description (10). [1-^11^C]acetate PET/CT and ^1^H-MRSI acquisition and analysis protocols are provided in *SI Appendix*, with the [1-^11^C]acetate production method detailed previously (12).

## Supplementary Material

Appendix 01 (PDF)

## Data Availability

Some study data are available. Access to the de-identified data can be provided upon a reasonable request to the corresponding author and is subject to appropriate regulatory approvals and data transfer agreements between the Cambridge University, Cambridge University Hospitals NHS Foundation Trust, and the recipient institution.
