# Supplementary material for: Spatial metabolomics informs the use of clinical imaging for improved detection of cribriform prostate cancer
Source: Proc Natl Acad Sci U S A. 2025 Jun 23;122(26):e2502423122. doi: 10.1073/pnas.2502423122 (PMC12232551; doi:10.1073/pnas.2502423122)
Supplement: Supplementary file 1 — Appendix 01 (PDF) [file pnas.2502423122.sapp.pdf]

Supplementary Information for

Spatial metabolomics informs the use of clinical imaging for improved detection of cribriform prostate cancer

Nikita Sushentsev[1*†], Gregory Hamm[2†], Roido Manavaki[1], Mary A. McLean[1], Jonathan Birchall[1], Dmitry Soloviev[3], David Y. Lewis[3], Luigi Aloj[1], Aleksandr Zakirov[4], Ian G. Mills[5,6,7], Vincent J. Gnanapragasam[8,9], Anne Y. Warren[10], Simon T. Barry[11], Richard J. A. Goodwin[2††], Ferdia A. Gallagher[1††], Tristan Barrett[1††]

[1] Department of Radiology, Addenbrooke's Hospital and University of Cambridge, Cambridge Biomedical Campus, CB2 0QQ, Cambridge, United Kingdom

[2] Integrated BioAnalysis, Clinical Pharmacology & Safety Sciences, R&D, AstraZeneca, Cambridge, UK

[3] School of Cancer Sciences, University of Glasgow, Glasgow, UK

[4] Department of Clinical Neurosciences, University of Cambridge, Cambridge, UK

[5] Nuffield Department of Surgical Sciences, University of Oxford, Oxford, UK

[6] Patrick G. Johnston Centre for Cancer Research, Queen's University Belfast, Belfast, UK

[7] Department of Biomedicine, Aarhus University, Aarhus, Denmark

[8] Department of Urology, Cambridge University Hospitals NHS Foundation Trust, Cambridge, UK

[9] Cambridge Urology Translational Research and Clinical Trials Office, Cambridge Biomedical Campus, Addenbrooke's Hospital, Cambridge, UK

[10] Department of Pathology, Cambridge University Hospitals NHS Foundation Trust, Cambridge, UK

[11] Bioscience, Discovery, Oncology R&D, AstraZeneca, Cambridge, UK

* **Correspondence**: Dr Nikita Sushentsev, **email:** ns784@medschl.cam.ac.uk

† These authors contributed equally to this project.

†† These authors jointly supervised this work.

This PDF file includes:

Supplementary text
SI references

Extended Methods

Spatial metabolomics study

The DESI-MSI protocol, along with its validation against MALDI-MSI and MS/MS techniques, has been described previously (1). To perform the metabolic pathway enrichment analysis (MPEA), the enriched metabolites (*P* > 0.05; Log2FC > 1) in either of the comparison groups were subjected to overrepresentation analysis. Only the pathways with an overall size of more than two metabolites, and having more than two enriched metabolites contributing to the named pathway (or "hits"), were retained. The produced output of MPEA contains a *P* value based on a hypergeometric test, as well as the pathway enrichment score (PES) for each pathway. PES represents a log2 transformed ratio between the number of observed enriched metabolites in the dataset and the number of metabolites expected to be enriched by random chance. In addition to the analysis of pathways as a whole (presented in **Figure 1** of the main manuscript), we conducted a similar analysis at the level of constituent metabolites, shown in the **Supplementary Figure 1** below.

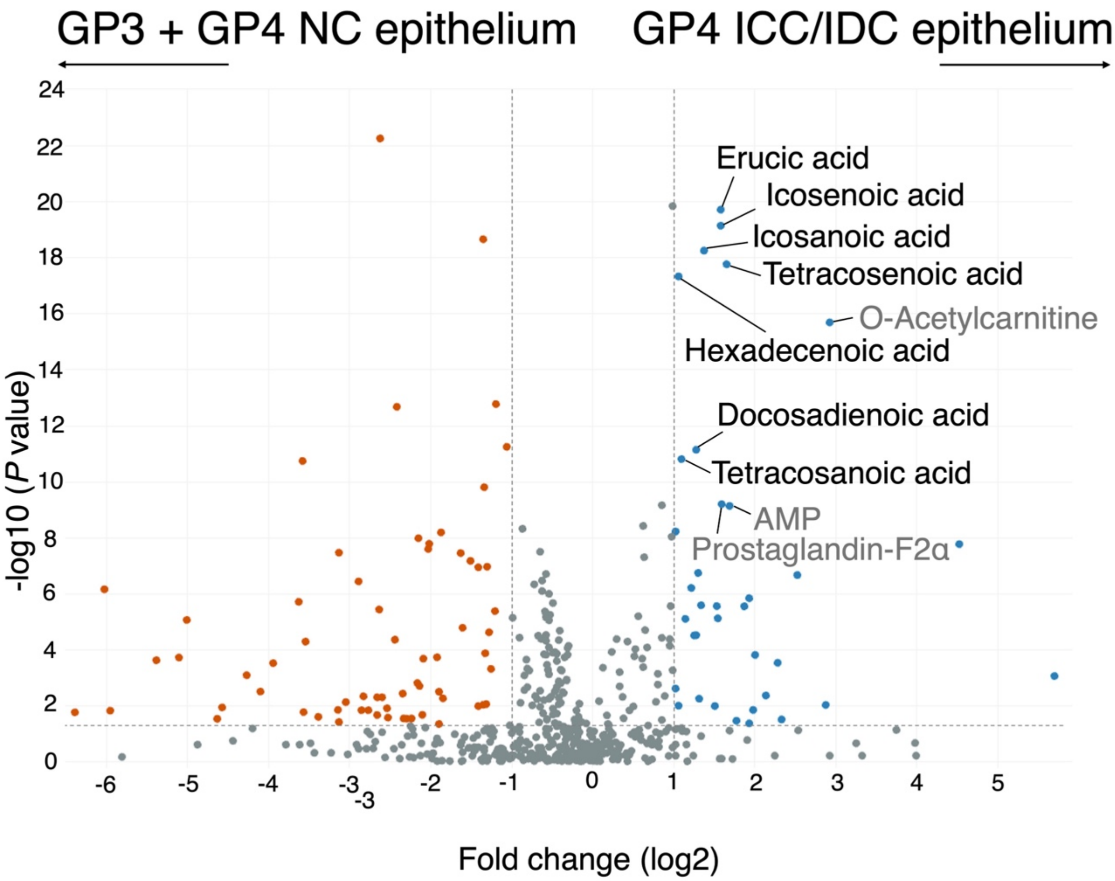

Supplementary Figure 1. Metabolic pathway enrichment analysis at the level of individual metabolites. This volcano plot demonstrates that of the ten most significantly enriched metabolites in cribriform epithelium, seven were unsaturated fatty acids.

[1-^{11}C]Acetate PET/CT study

Patient characteristics

9 patients with biopsy-proven intermediate- or high-risk prostate cancer were prospectively enrolled and underwent a dedicated research PET/CT examination prior to treatment with radical prostatectomy with written informed consent obtained in all cases. Approval for the study was granted by the institutional review board, the local ethics committee (CUH/15/EE/0213), and the Administration of Radioactive Substances Advisory Committee (ARSAC).

PET/CT imaging

Subjects were scanned supine using a GE Discovery 690 (GE Healthcare, Waukesha, WI, US) at Addenbrooke's Hospital, Cambridge, UK. Immediately following a low-dose CT of the pelvis for attenuation correction and anatomical localisation, patients received an intravenous bolus injection of 1.22 ± 0.19 GBq ^{11}C-acetate. Emission data were acquired in list-mode over 90 minutes in one bed position with the prostate centred axially in the field-of-view (FOV). List-mode data were binned in 54-time frames of increasing duration (12×5 s, 3×10 s, 6×15 s, 4×30 s, 5×60 s, 5×120 s, 10×150 s, 9×300 s) and reconstructed into a 192x192x47 matrix with 3.12×3.12×3.27 mm voxels, using time-of-flight ordered-subsets expectation maximisation (TOF-OSEM) with 4 iterations and 24 subsets. Corrections for attenuation, scatter, randoms, dead time, normalisation, sensitivity and isotope decay were applied as implemented on the scanner, together with an isotropic 4-mm full-width at half maximum Gaussian filter post reconstruction. The attenuation correction CT (CT-AC) acquisition parameters were: tube voltage 140kV, tube current 80mA, rotation time 0.5 s, pitch 1.375, slice thickness 3.75 mm reconstructed to 3.27 mm. ^{11}C-acetate was provided by the Wolfson Brain Imaging Centre, Cambridge, UK.

Participants were not required to fast prior to their PET/CT examination. To capture exhaled ^{11}C-CO_2, the main metabolite of ^{11}C-acetate in blood, Subjects wore an oral-nasal face mask connected to a closed, valve-free tubing system. This system utilised a continuous flow pump operating at ~20 L/min to direct exhaled air through a medical-grade double soda lime CO_2 absorber (Spherasorb™, Intersurgical, Wokingham, UK). Considering a respiratory rate of 12 breaths/min, a tidal volume of ~0.5 L and 4% CO_2 concentration in exhaled air, the system was designed to capture 21.6 L of CO_2 from exhalation and an additional 0.72 L from room air (0.04% CO_2 concentration), requiring 186 g of soda lime for full absorption.

Magnetic resonance imaging

All participants underwent prostate MRI examinations as part of their clinical management, performed at 1.5 T or 3.0 T scanners using a surface coil and no endorectal coil. All studies met minimum PI-RADS sequence requirements and included axial T_1-weighted images of the pelvis, high resolution axial T_2-weighted images of the prostate and DWI with a minimum of two b values, with a high b value of ≥800-1,000. To avoid bias in selection of regions with high uptake on PET, the T_2-axial MR images and whole-mount pathology slides were utilized for the definition of ROIs for use in the analysis of the PET data.

Image analysis

PET, CT and MR image registration: Image registration was performed blinded to the clinical data of the participants and utilised models implemented); Penn Image Computing and Science Lab, University of Pennsylvania, USA). The CT-AC image of each ^{11}C-acetate examination was used as the reference space for all registrations performed in this study.

To reduce the impact of patient motion during the ^{11}C-acetate PET acquisition, dynamic PET image series were non-rigidly registered to the time frame corresponding to 55-60 min post tracer injection using the Advanced Normalization Tools (ANTs) package (https://stnava.github.io/ANTs/). Registration utilised a 4-level multi-scale approach with affine initialisation, employing normalised cross-correlation as the similarity metric. Adjustment for local non-rigid deformations was performed using symmetric normalisation (SyN) with Gaussian regularisation (3 voxels) of the update velocity fields and zero total field regularisation. The registered PET frames were averaged to generate a mean image, which was subsequently co-registered with the CT-AC using affine transformation and mutual information similarity. PET images were co-registered with the T_2-weighted MRI through the CT-AC, serving as the intermediate link for spatially registering the datasets. As above, T_2-weighted images were initially registered to the CT-AC using affine transformation, followed by non-rigid registration using SyN and mutual information as the similarity metric. To avoid the registration process being influenced by pelvic bone anatomy and structures distant to the prostate, a sub-volume encompassing the prostate gland alone were utilised. The quality of the results obtained by the registration process was visually inspected. The resulting affine and non-rigid transformations were inverted and subsequently applied to the motion-corrected PET dynamic series to achieve co-registration with the T_2-weighted MRI.

Lesion-based analysis: Registered frames of the dynamic PET image series corresponding to 6-10 min, 14-20 min, 20-30 min and 60-90 min post injection were averaged, and utilised for the generation of standardised uptake value (SUV) images normalised by body weight (SUV_{bw}). Tumour regions were delineated on contiguous slices of the clinical diagnostic MRI, encompassing the entire lesion, by a radiologist with 13-years' experience reporting clinical prostate MRI. Regions for benign prostate tissue were also delineated. The demarcated regions were transposed onto the PET SUV maps, co-registered with the T_2-weighted images, enabling the calculation of SUV_{mean} and SUV_{max} within these regions. Tissue time activity curves over the 90-min PET acquisition were derived by applying these regions onto the registered PET dynamic series.

Histopathological correlation

Prostatectomy specimens were fixed in formalin and oriented by the location of the seminal vesicles, posterior surface of the prostate, and by the position of the urethra. The apical cone was amputated and sliced into 4 mm sections from left to right, the remaining gland was cut transversely into 5 mm whole-mount parallel slices in the horizontal plane from inferior to superior; the base was sliced in a similar manner to the apex. Tumor was outlined on hematoxylin and eosin (H&E) stained sections from each slice by an experienced uropathologist specializing in prostate cancer (A.Y.W.). Each histopathology slice was manually co-registered to the corresponding PET/CT slice, with utilization of the diagnostic MR images where necessary. 2019 International Society of Urological Pathology (ISUP) consensus recommendations were used (2) to record the global Gleason score and corresponding grade group, along with the presence of intraductal carcinoma (IDC). Patients who had either invasive cribriform carcinoma (ICC) or IDC or both according to the ISUP consensus definition of cribriform PCa (3) were assigned a "cribriform-positive" label; patients who had neither morphology were assigned a "non-cribriform" label.

^{1}H-MRSI study

Patient characteristics

12 patients with biopsy-proven intermediate-risk prostate cancer were prospectively enrolled between June and August 2024 and underwent a dedicated research ^{1}H-MRSI examination added to their standard-of-care biparametric prostate MRI protocol, which was described previously (4).

^{1}H-MRSI acquisition and analysis

Following the acquisition of the clinical biparametric MRI sequences, all patients underwent ^{1}H-MRSI across a 4.5 mm thick axial slice through the tumour (Hamming-filtered density weighted trajectory over 3231 points of k-space, FOV 32 cm, matrix 64x64 interpolated to 128x128, 30° flip, TR 88.7 ms, TE 2.1 ms, 5 kHz full-receiver bandwidth, 256 spectral points), using the Multinuclear Research Package (GE Healthcare, Waukesha WI, USA). The original spectra, as obtained during image acquisition, are presented in **Supplementary Figure 2.** The slice showing the target lesion in its largest axial diameter was selected based on anatomical T2WI and DWI by a radiologist with 13-years' experience reporting clinical prostate MRI. Image acquisition time for the single-slice ^{1}H-MRSI was 4 min and 47 s. Maps of the ratio of fat to fat + water (F/F+W) were calculated from MRSI as follows, using custom code developed in Matlab R2023b (Mathworks, Natick, MA, USA). Magnitude spectra were first corrected for bias by subtraction of the mean value between 19.5-21.0 ppm in a region outside the body, then integrated over the ppm ranges 3.0 – 6.1 (water) and -0.2 – 2.9 (fat) and used to calculate Fat / (Fat + Water). The resulting ratios, with value between 0 and 1, were multiplied by 1000 and the resulting maps were written as DICOM images using header information from the original MRSI series; the maps, along with the thickened spectra reproduced in black for illustrative purposes, are presented in **Supplementary Figure 3.** Manual registration of Fat / (Fat + Water) DICOM images with the anatomical T2WI MR images was performed using ITK-SNAP where necessary to compensate for the potential movement of the prostate between the anatomical T2WI and ^{1}H-MRSI acquisition. ROIs encompassing the areas of histologically confirmed tumour and benign areas were then drawn by a radiologist with 13-years' experience reporting clinical prostate MRI as shown in **Figure 2** of the main manuscript, with the resulting ratios used for the comparisons presented in the main manuscript.

Histopathological correlation

All patients underwent targeted transperineal biopsies (Biopsee, Oncology Systems Limited), which included the sampling of 24 systematic cores and 2-4 separate target cores as described previously (5). Biopsy specimens were reviewed by an expert genitourinary pathologist (A.Y.W.), with their location used to guide ^{1}H-MRSI segmentation as shown in **Fig. 2** of the main manuscript. Tumor grading and identification of cribriform disease were done as described above.

Statistics and reproducibility

Statistical analyses were conducted using GraphPad Prism (version 10.4.1, GraphPad Software, San Diego, CA, USA). Normal distribution of the data was assessed using the D'Agostino-Pearson test (threshold $P \geq 0.05$). A two-tailed Mann-Whitney *U* test was used to assess the intergroup differences in ^{1}H-MRI ADCratio and epithelial cell density. A one-tailed Mann-Whitney *U* test was used to assess the intergroup differences in other metrics as our hypothesis based on the spatial metabolomics analysis was that cribriform tumors would demonstrate higher [1-^{11}C]acetate uptake and ^{1}H-MRSI fat fraction due to upregulated fatty acid metabolism. *P* values below 0.05 were considered significant. All experiments were independent and standalone.

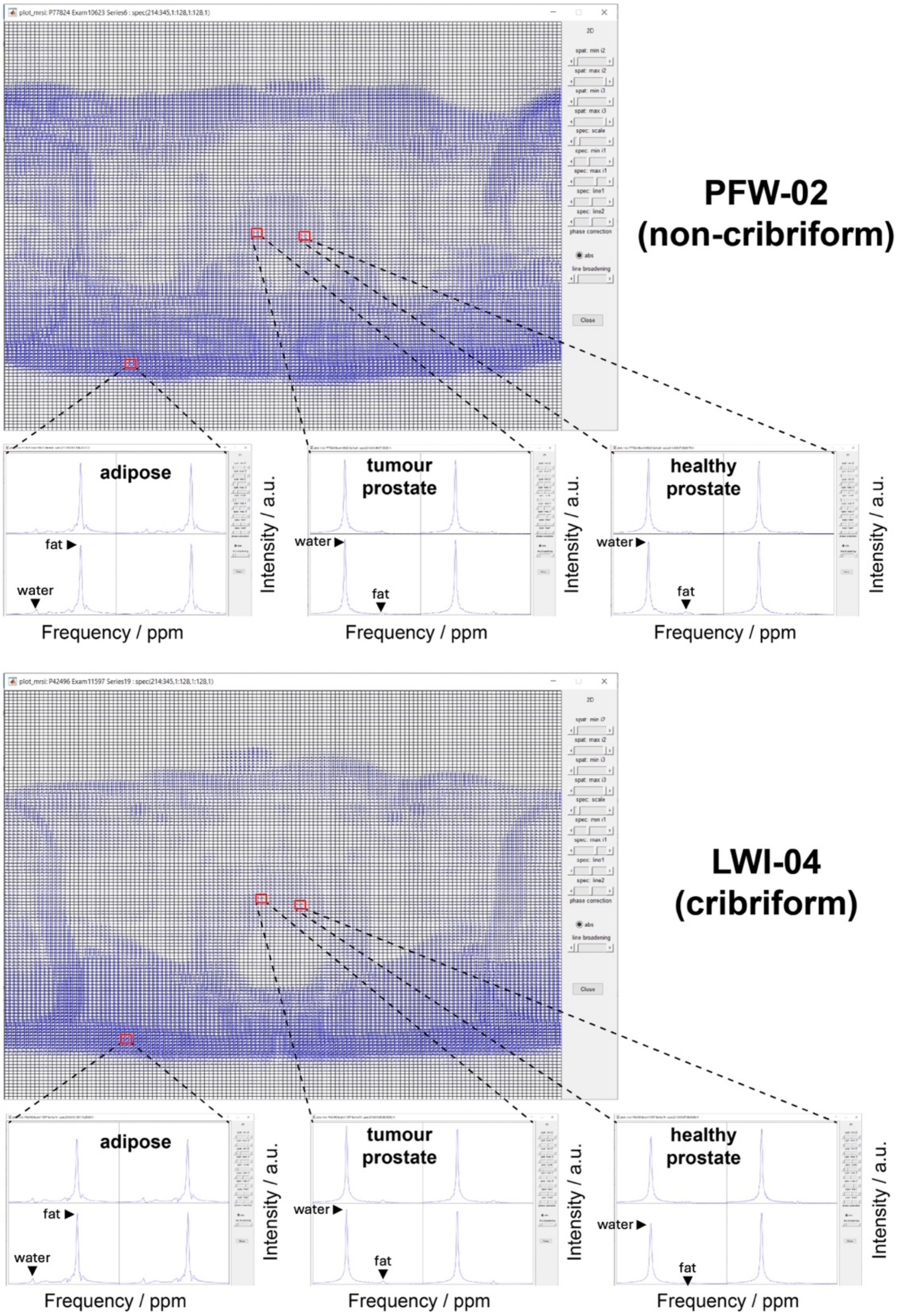

Supplementary Figure 2. Raw MRSI data obtained during image acquisition. Full pelvic coverage using ^{1}H-MRSI, with the output presented using the default scanner reconstruction. Three regions-of-interest (subcutaneous fat, benign prostate, malignant prostate) are denoted, with the underlying raw spectra presented in 2x2 boxes (note thin lines and default blue color).

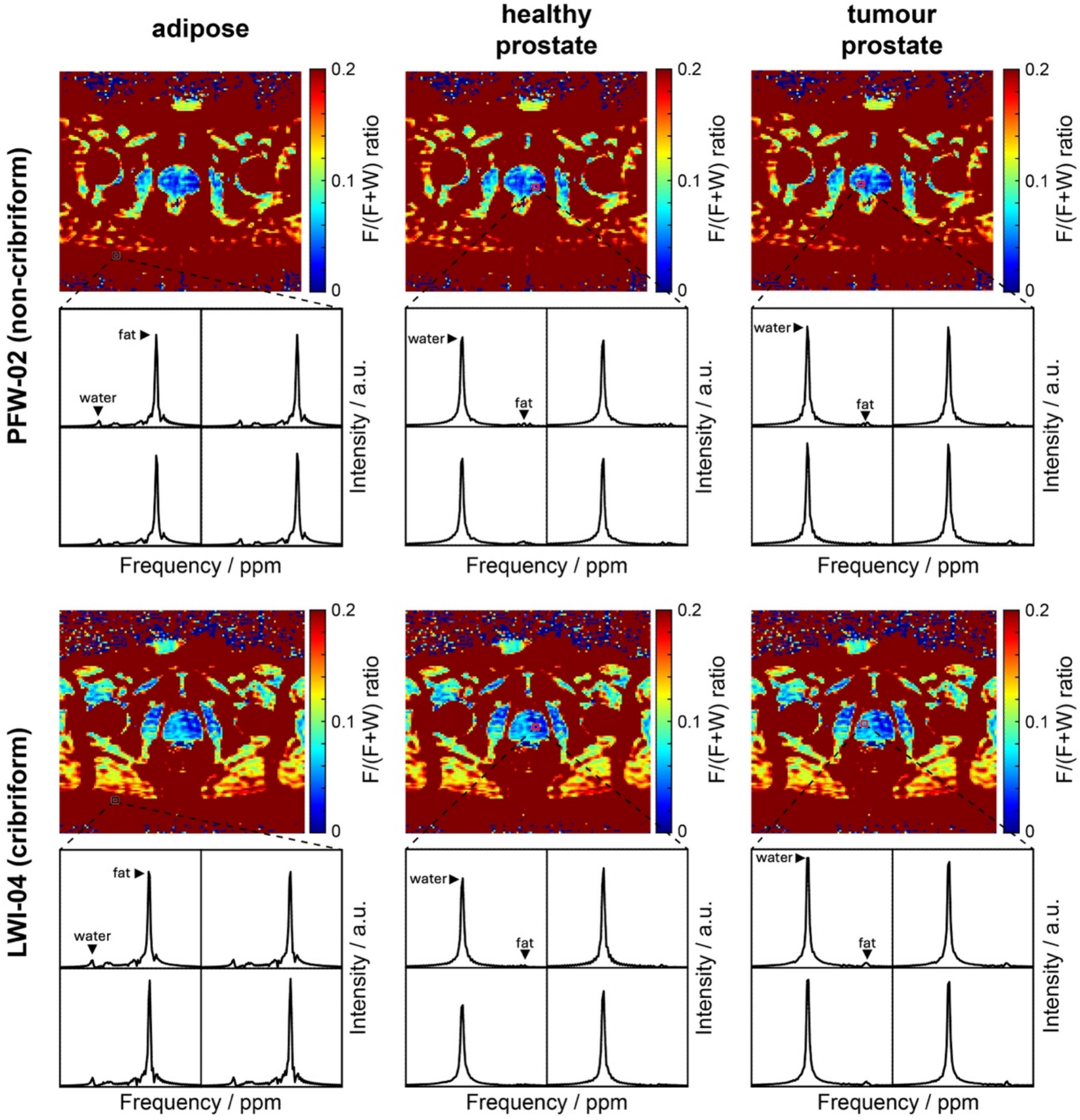

Supplementary Figure 3. Fat fraction maps with representative spectra. Fat fraction maps are shown for the same patients as presented in **Supplementary Figure 2** and **Figure 2** of the main text. Representative spectra from the same regions-of-interest (subcutaneous fat, benign prostate, malignant prostate) are the same raw spectra presented in in **Supplementary Figure 2**, with the only changes being line thickening and black coloring to aid visualisation in **Figure 2** of the main text. The extensive red in the heatmap results from setting an upper threshold of 0.2 on the jet colorscale used, in order to better visualize variation in fat fraction across the prostate.

Data sharing plans

Access to the de-identified data can be provided upon a reasonable request to the corresponding author and is subject to appropriate regulatory approvals and data transfer agreements.

Supplementary references:

1. N. Sushentsev, *et al.*, Metabolic imaging across scales reveals distinct prostate cancer phenotypes. *Nat Commun* **15** (2024).
2. Van Leenders, G. J. L. H. *et al.* The 2019 International Society of Urological Pathology (ISUP) Consensus Conference on Grading of Prostatic Carcinoma. *Am J Surg Pathol* **44**, E87–E99 (2020).
3. Van Der Kwast, T. H. *et al.* ISUP Consensus Definition of Cribriform Pattern Prostate Cancer. *Am J Surg Pathol* **45**, 1118–1126 (2021).
4. N. Sushentsev, *et al.*, The effect of capped biparametric magnetic resonance imaging slots on weekly prostate cancer imaging workload. *Br J Radiol* **93** (2020).
5. T. Barrett, *et al.*, Three-year experience of a dedicated prostate mpMRI pre-biopsy programme and effect on timed cancer diagnostic pathways. *Clin Radiol* **74**, 894.e1-894.e9 (2019).
